# Mediastinal lipomatosis in a patient with Bardet-Biedl syndrome: more diverse than previously thought

**DOI:** 10.11604/pamj.2023.45.82.35582

**Published:** 2023-06-13

**Authors:** Eleni Paschou, Nikolaos Sabanis

**Affiliations:** 1 Department of General Practice and Family Medicine, Health Center of Aliartos, Levadia, Greece,; 2 Department of Nephrology, General Hospital of Trikala, Trikala, Greece

**Keywords:** Bardet-Biedl Syndrome, mediastinal lipomatosis, ciliopathies, chronic kidney disease, renal hypoplasia, brachyctyly

## Image in medicine

A 52-year-old patient with end-stage renal disease (ESRD), undergoing intermittent hemodialysis for 20 consecutive years was recently diagnosed with Bardet-Biedl syndrome (BBS). His medical history included central obesity, hypogonadism, blindness due to pigmented retinopathy, neurological disorders (epilepsy, ataxia, peripheral neuropathy), learning disabilities, speech disorders, brachydactyly, and ESRD attributable to bilateral renal hypoplasia. A chest computed tomography was performed due to shortness of breath and revealed mediastinal lipomatosis (ML). ML is usually a benign and asymptomatic intrathoracic condition. It is associated with steroid use, obesity, hyperlipidemia, diabetes mellitus, metabolic syndrome, or Cushing syndrome. Occasionally it becomes symptomatic, manifesting with breathlessness, dry cough, dyspnea, thoracic pain, or supraventricular tachycardia. To the best of our knowledge, this is the first case of ML in a patient with BBS. Bardet-Biedl syndrome (BBS) is a rare multisystem autosomal recessive disease associated with disorders in the structure and function of primary cilia, characterized by high genotypic and phenotypic heterogeneity. Its prevalence is 1-9/1.000.000 in Western countries. Depending on the population origin, BBS1 and BBS10 are the most frequent genes, accounting for ~23% and 15% of genotyped BBS patients respectively. The diagnosis is based on the presence of four major criteria (pigmented retinopathy, polydactyly, chronic kidney disease (CKD), hypogonadism, mental retardation, central obesity) or three major and two minor criteria (brachydactyly/syndactyly, dental dysnormalities, anosmia, taste impairment, neuropathy, liver fibrosis, diabetes mellitus, congenital heart disease) confirmed by genetic testing. In BBS, the main cause of morbidity and mortality is CKD; its severity varies among patients leading potentially to ESRD requiring dialysis or transplantation.

**Figure 1:**
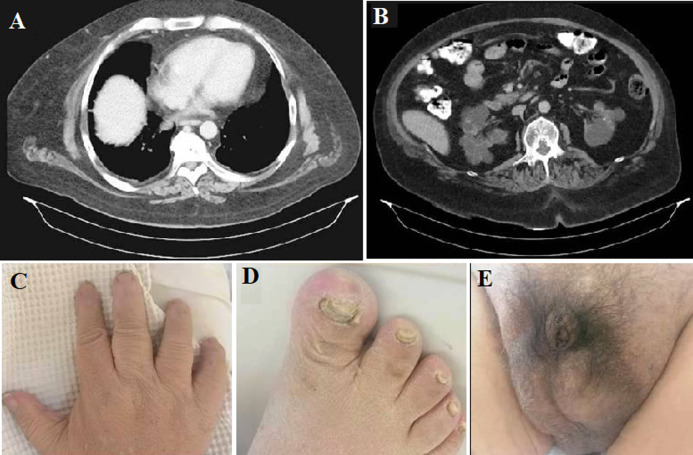
computed tomography findings and clinical characteristics of a 52-year-old patient with Bardet-Biedl syndrome; A) mediastinal lipomatosis; B) renal hypoplasia; C) brachydactyly of fingers; D) brachydactyly of toes; E) hypogonadism (micropenis)

